# Resting-state hemodynamic changes and effects on upper limb function after multi-channel transcranial direct current stimulation to the ipsilesional primary motor cortex and anterior intraparietal sulcus in stroke patients: an fNIRS pilot study

**DOI:** 10.1186/s12984-025-01618-8

**Published:** 2025-04-16

**Authors:** Seung Hyun Lee, Gihyoun Lee, Jinuk Kim, Zephaniah Phillips V, Heegoo Kim, Eunmi Kim, Su-Hyun Lee, Ho Choon Jeong, Seung-Ho Paik, Yun-Hee Kim, Beop-Min Kim

**Affiliations:** 1 Global Health Technology Research Center, College of Health Science, Korea University, Seoul, Republic of Korea; 2 School of Biomedical Engineering, Chonnam National University, Yeosu, Republic of Korea; 3 Center for Neuroscience Imaging Research, Institute for Basic Science (IBS), Suwon, Republic of Korea; 4 Department of Physical and Rehabilitation Medicine, Sungkyunkwan University School of Medicine, Suwon, 16419 Republic of Korea; 5 Department of Rehabilitation Medicine, Department of Rehabilitation Medicine, CHA University School of Medicine, Seongnam, Republic of Korea; 6 Digital Therapeutics Research Team, CHA Bundang Medical Center, CHA Future Medicine Research Institute, CHA University School of Medicine, Seongnam, Republic of Korea; 7 CyberMedic Co., Ltd, Gwangju, Republic of Korea; 8 KLIEN Inc, Seoul, Republic of Korea; 9 Myongji Choonhey Rehabilitation Hospital, Seoul, Republic of Korea; 10 Department of Biomedical Engineering, Korea University, Seoul, 02841 Republic of Korea

**Keywords:** Transcranial direct current stimulation, Functional near-infrared spectroscopy, Stroke, Cerebral oxygenation, Motor cortex, Brain connectivity

## Abstract

**Background:**

Stroke results in substantial long-term disability, necessitating effective recovery interventions. This study explored the effects of multi-channel transcranial direct current stimulation (tDCS) on hemodynamic responses and upper limb motor function in stroke patients, targeting the ipsilesional primary motor cortex (M1) and anterior intraparietal sulcus (aIPS).

**Methods:**

A double-blind, randomized, sham-controlled trial was conducted with 24 stroke patients (18 men; mean age, 57.3×14.2 years), who underwent 10 sessions of real or sham multi-channel tDCS combined with upper limb exercises. Functional near-infrared spectroscopy (fNIRS) measured resting-state cerebral hemodynamic responses for 5 min before and after each session. Motor function was evaluated using the Fugl–Meyer assessment for upper extremity (FMA-UE), box and block test (BBT), and other motor function tests before and after the interventions.

**Results:**

The real multi-channel tDCS group exhibited increases in regional accumulation of oxyhemoglobin (HbO_Acc_) and stronger seeded connectivity networks within the motor cortex poststimulation. In contrast, the sham group exhibited disassociation from these areas. The group × time interaction was significant for the Box and Block Test (BBT), indicating greater improvements in gross manual dexterity in the real-tDCS group compared to the sham group. While poststimulation changes in HbOAcc were examined in relation to FMA-UE scores, no strong linear relationship was observed in the real-tDCS group.

**Conclusions:**

Multi-channel tDCS targeting the ipsilesional M1 and aIPS, combined with upper limb exercises, showed potential effects on cerebral hemodynamics and motor function in stroke patients. These findings suggest that multi-channel tDCS may have a role in motor rehabilitation, but further research is needed to validate its efficacy and clinical applicability.

**ClinicalTrials.gov:**

This study was registered at ClinicalTrials.gov (NCT05275114).

**Supplementary Information:**

The online version contains supplementary material available at 10.1186/s12984-025-01618-8.

## Background

Stroke, a leading cause of long-term disability worldwide, presents substantial challenges in the recovery of motor function [1, 2]. Transcranial direct current stimulation (tDCS) has emerged as a promising noninvasive brain stimulation technique that modulates neuronal excitability, potentially enhancing motor learning and aiding in poststroke recovery [3, 4]. The synergy of tDCS with conventional rehabilitation therapies has been increasingly explored, revealing a promising avenue for accelerating motor recovery [5].

Recent technological advancements have introduced multi-channel tDCS, an innovative approach utilizing smaller, multiple electrodes to offer precise stimulation [6]. The precise, localized stimulation capability of multi-channel tDCS presents a novel opportunity to explore additional beneficial brain areas for motor recovery, potentially enhancing therapeutic outcomes [7, 8]. This method allows for the targeted activation of crucial motor recovery areas, including the primary motor cortex (M1), extending beyond the conventional scope [9, 10]. The parietal lobe plays a critical role in the early stages of motor recovery, particularly in acquisition and consolidation, by closely coordinating with attention-related functions of the frontal lobe [11]. The anterior intraparietal sulcus (aIPS) within the parietal lobe has been identified as a significant region correlated with motor functions in both healthy individuals and stroke research [12, 13]. Leveraging multi-channel tDCS devices capable of high spatial resolution stimulation to target not only the ipsilesional M1 but also the aIPS presents a promising avenue for enhancing upper limb and hand motor function recovery in stroke patients.

Understanding the mechanisms underlying tDCS-induced motor recovery is crucial. To this end, neuroimaging modalities, such as functional magnetic resonance imaging (fMRI) have been utilized to observe changes in brain activity resulting from tDCS [14, 15]. In this context, functional near-infrared spectroscopy (fNIRS) emerges as a pivotal tool providing insights into cerebral hemodynamic responses to tDCS [16]. The integration of fNIRS enables real-time monitoring of brain activity changes, shedding light on the neural correlates of functional recovery. This enhances our understanding of the therapeutic mechanisms of tDCS [17, 18]. Previous studies on tDCS-fNIRS in healthy participants and patients with stroke have revealed high interparticipant variability and/or inconclusive results [19]. This might be attributed to the lack of a standardized metric to accurately assess changes in cerebral oxygenation [16]. Given the multitude of studies focusing on measuring resting-state cerebral responses, conventional task-evoked metrics, such as minimum, maximum, and mean values, are unsuitable for quantifying these brain responses. Instead, a previous study has demonstrated that integrating time-series hemodynamic data provides a reliable marker of accumulated cerebral oxygenation, capable of identifying patients with autonomic response impairments [20]. Quantifying the accumulation of oxyhemoglobin (HbO_Acc_) and deoxyhemoglobin (Hb_Acc_) enables a more accurate capture of the net change in cerebral oxygenation induced by tDCS in a resting state.

In this pilot study, we utilize the high spatial resolution of multi-channel tDCS devices to focus precisely on the ipsilesional M1 and aIPS in patients with stroke. This study aims to assess the effects of such targeted stimulation on measuring changes in cerebral hemodynamic responses and improving upper limb and hand motor functions. By integrating an multi-channel tDCS-fNIRS system, this study aims to measure the changes in resting-state hemodynamic responses before and after stimulation. It also seeks to identify cerebral oxygenation changes associated with tDCS-induced improvements in motor function. We hypothesize that multi-channel tDCS will have significant effects on upper limb motor function and modulate hemodynamic responses in stroke patients. In contrast, no significant changes are expected in the sham condition. Through this focused investigation, we aim to contribute to the neurorehabilitation field by providing insights into stroke recovery strategies and the mechanisms behind motor function enhancement.

## Methods

### Participants

Potential participants were recruited from an outpatient stroke rehabilitation clinic at Samsung Medical Center in Seoul, Republic of Korea, between March 2022 and August 2022. Participants who signed the informed consent form for the study were screened by a rehabilitation medicine specialist to ensure their eligibility according to the study’s inclusion criteria. The final analysis included 24 participants (18 men; mean age, 57.3 ± 14.2 years) out of the original 28 who had initially given their consent (Supplementary Fig. 1). This exclusion occurred because two participants failed to meet the inclusion criteria, one withdrew consent before the experiment, and another discontinued the intervention. Among the participants who completed the study, a significant difference in stroke type was observed between the groups. However, no significant differences were observed in other characteristics, such as age, sex, time since stroke onset, lesion side, or initial FMA-UE scores (Table 1).

**Table 1:** Demographic characteristics of the participants

	Real-tDCS (*n* = 12)	Sham-tDCS (*n* = 12)	*p*-value
Age (years)	52.3±15.5	58.5±16.41	0.084
Sex (male: female)	9: 3	9: 3	1.000
Time since stroke onset (months)	60.3±50.9	38.8±25.6	0.205
Lesion side (right: left)	3: 9	4: 8	0.653
Stroke type (infarction: hemorrhage)	6: 6	11: 1	0.025^*^
Initial FMA-UE score	44.3±12.1	37.8±18.4	0.590

Values are presented as the mean ± standard deviation. tDCS, transcranial direct current stimulation; FMA-UE, Fugl–Meyer assessment for Upper Extremity: ^*^, Significant difference between groups, *p* < 0.05

The study recruited participants aged between 19 and 80 years who had experienced a stroke at least three months before their enrollment. To be eligible, participants were required to have a unilateral lesion, excluding those in the M1 and the aIPS, which are the areas of stimulation. Furthermore, they were required to demonstrate moderate to severe impairment in upper extremity function, as determined by a Fugl–Meyer assessment for upper extremity (FMA-UE) score of less than 58 [21]. Exclusion criteria for the study were as follows: participants with significant neurological conditions other than stroke; individuals with major psychiatric disorders, such as schizophrenia or bipolar disorder; and those with cognitive impairments that could impede participation in the study were excluded. Participants who had received botulinum toxin injections or nerve block procedures within 6 months before consenting, or who had undergone surgical treatment for the peripheral nerves, muscles, or tendons of the upper limb, were also excluded. Furthermore, individuals deemed unsuitable for tDCS due to having implanted electronic medical devices (e.g., pacemakers), metal objects in the skull, skin lesions at the attachment site, or being pregnant or breastfeeding were excluded [22]. This study was conducted by the Declaration of Helsinki and was approved by the Institutional Review Board (IRB) of Samsung Medical Center, Seoul, Republic of Korea (IRB No. 2021-07-176), and was registered at ClinicalTrials.gov (NCT05275114).

### Study design

**Fig. 1:**
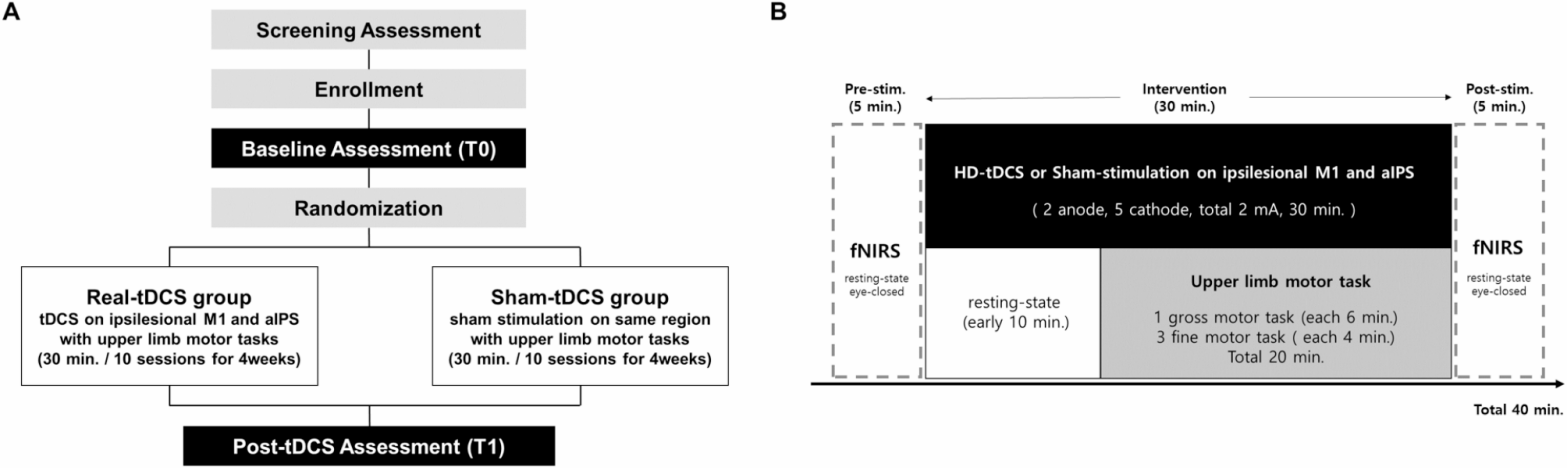
**(A)** Study design **(B)** Intervention protocol. Each intervention entailed a total of 30 min of multi-channel tDCS application, starting with 10 min of resting-state stimulation followed by 20 min of upper limb motor tasks alongside continued stimulation. fNIRS measurements were conducted during an eyes-closed resting state for 5 min before and after each intervention

This study was designed as a single-center, double-blind, randomized, sham-controlled trial. Enrolled participants were randomly assigned to either the real-tDCS group or the sham-tDCS group using a predetermined randomization table, maintaining a 1:1 allocation ratio (Fig. 1A). Each participant engaged in upper limb motor exercises while receiving multi-channel tDCS aimed at activating the ipsilesional M1 and aIPS areas, as per the assigned tDCS condition. The intervention was administered once daily for 30 min, up to three times a week, for a total of 10 sessions over four weeks. For the initial 10 min of the 30-minute intervention period, only tDCS was applied, followed by a combination of tDCS and upper limb motor exercises for the remaining 20 min (Fig. 1B). The exercises included one gross motor task (such as the figure-8 exercise, shoulder arc ROM exercise, or stacking cones) for 6 min, followed by three fine motor tasks (such as picking up small objects, using a pinch clip, writing, card turning, the Purdue pegboard task, or putty-exercise) for 4 min each, totaling 20 min. Tasks were tailored to the participant’s functional level by a licensed physical therapist. To evaluate changes in regional hemodynamic responses to multi-channel tDCS, fNIRS measurements were conducted for 5 min before and after each intervention session, with participants in an eyes-closed resting state. Upper limb functional assessments were conducted before (T0) the intervention commenced and after completing all 10 interventions (T1) to evaluate the improvement in upper limb function.

### Integrated multi-channel tDCS and fNIRS device

**Fig. 2:**
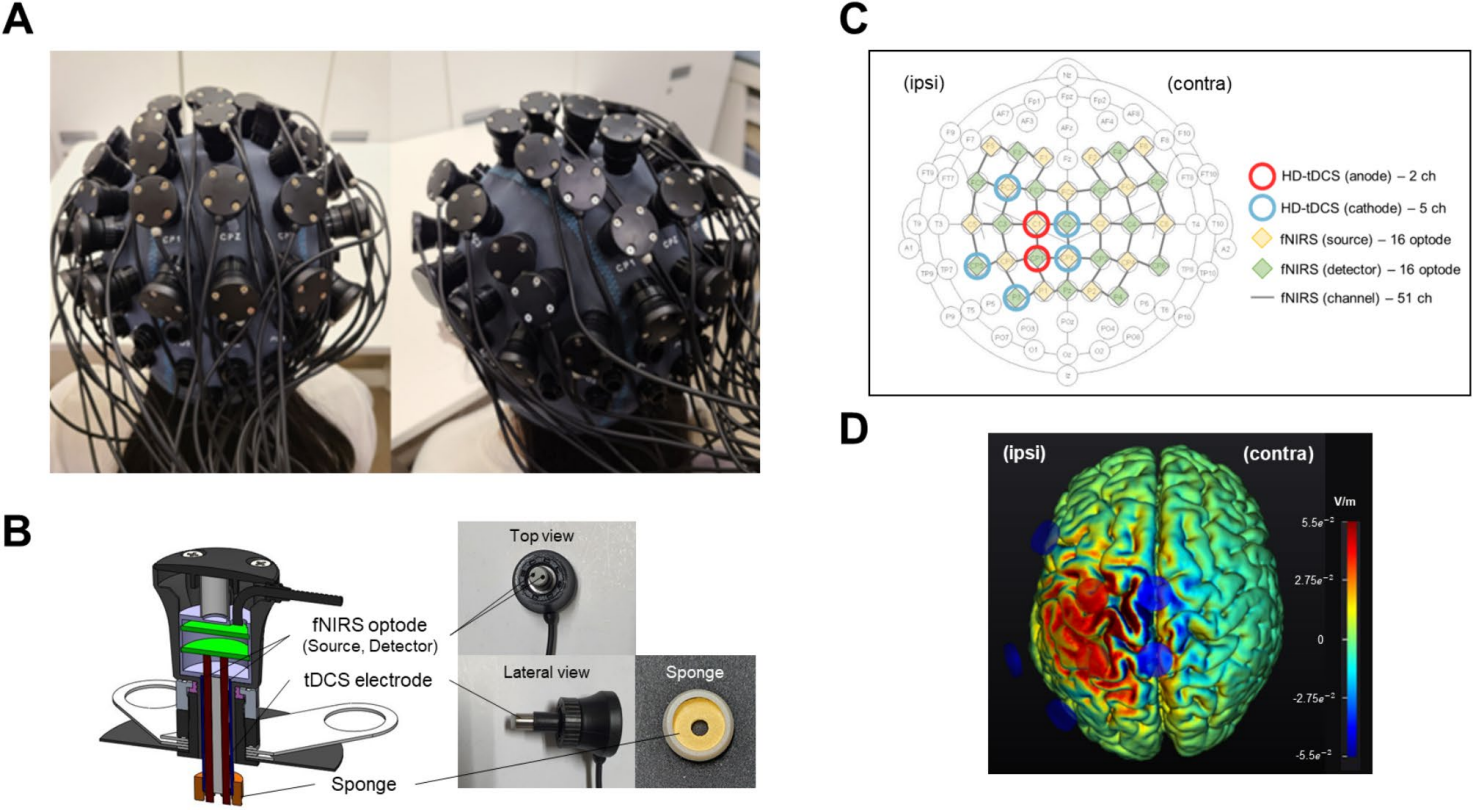
Integrated multi-channel transcranial direct current stimulation and functional near-infrared spectroscopy (fNIRS) system. **(A)** Participants wearing the integrated device. **(B)** Close-up view of an multi-channel tDCS-fNIRS module. The integrated module comprises a pair of fNIRS optodes (one source and one detector) and a tDCS electrode. The tDCS electrode, coupled with a saline-soaked, ring-shaped sponge, administers current to the scalp. A rubber guide encircles the sponge, ensuring its stable placement. **(C)** Layout of the multi-channel tDCS-fNIRS system on the head. The system configuration is composed of 32 integrated modules, each containing two anodes and five cathodes for multi-channel tDCS, along with 16 sources and 16 detectors forming 51 fNIRS measurement channels. **(D)** Simulation results demonstrate the distribution of the normal electric field component (V/m) during multi-channel tDCS application using a standard anatomical template. The simulation was performed using the Neurophet tES LAB software (Neurophet, Seoul, South Korea). Red circles represent anodes, and blue circles represent cathodes. The positive values represent the inward electric field relative to the cortical surface, while negative values represent the outward electric field. The electric field concentration is notably strong around the M1 and aIPS regions, aligning with the anode placements

In this study, the NT Brain 100 device (CyberMedic Co., Ltd., Republic of Korea) was utilized to administer multi-channel tDCS and measure fNIRS (Supplementary Fig. 2). The device is equipped with an electrode for delivering electrical current, along with a light-emitting diode and photodetector integrated within a compact probe measuring 24(W) × 24(D) × 39(H) mm, specifically designed for measuring cerebral hemodynamic responses. This integrated system facilitates targeted stimulation and precise measurement of brain regions, streamlining the modulation and assessment of cerebral activity and vascular changes. Figure 2A illustrates the integrated multi-channel tDCS and fNIRS system attached to a participant’s head, along with a sample tDCS-fNIRS module (Fig. 2B), including a connector for data acquisition, a tDCS electrode, and an fNIRS source/detector fiber tip. Each module was secured to the head using a custom-designed cap, positioned according to the 10–20 system for optimal placement. The cap’s modular holder contains a soaked sponge to facilitate the conduction of electrical current from the tDCS electrode at the electrode-skin interface. Additionally, the module’s fiber tip is designed to be retractable, ensuring proper contact with the skin for precise measurements.

#### Multi-channel tDCS administration

In this study, the ipsilesional M1 and aIPS were identified as primary targets for multi-channel tDCS, aiming to enhance upper limb recovery in patients with stroke. The hand area of M1, adjacent to C1 in the left hemisphere [23], has been a focal point in previous tDCS studies due to its essential role in motor recovery [3]. Moreover, prior research has demonstrated the aIPS, situated near CP1 in the left hemisphere, is significantly associated with motor function recovery [12, 24]. Multi-channel tDCS precisely targeted the specified areas using seven ring-shaped electrodes, each with a diameter of 2 cm. In participants with left-sided lesions, anodal stimulation was applied at C1 and CP1, each receiving 1.0 mA. The arrangement also included cathodal electrodes placed around these anodal points with varying intensities: C1 and CP1 each receiving 1.0 mA for anodal stimulation; and for cathodal stimulation, Cz at − 0.95 mA, FC3 at − 0.25 mA, CPz at − 0.40 mA, CP5 at − 0.05 mA, and P3 at − 0.35 mA (Fig. 2C, D). For right-sided lesions, electrode placement was symmetrically adjusted to ensure an equal distribution of the total current from the anodal electrodes among the cathodes. The surface area of each electrode was 2.55 cm², resulting in a current density of 0.392 mA/cm² at 1 mA, a level deemed safe by previous studies [25]. To enhance participant comfort and safety, the stimulation included a 30-s ramping up and down period at the beginning and end.

For the sham-tDCS group, the procedure mimicked actual stimulation with 30 s ramping up and down periods. However, after the ramp-up and ramp-down phases, the device was turned off, ensuring that no active current was delivered for the duration of the session. This method simulated the sensation of stimulation without delivering active current, a common technique in tDCS research to ensure blinding and control for placebo effects [26].

#### fNIRS measurement

The fNIRS measurement setup in this study comprised 32 modules placed over the frontal, motor, and parietal areas of the brain (Fig. 2C). Continuous-wave fNIRS monitoring was employed, using light-emitting diodes alternating between 780 nm and 850 nm wavelengths at an output power of less than 2 mW. Light intensity changes were detected using a silicon photomultiplier. In each module, the source and detector fibers were positioned 5 mm apart, constituting short-separation channels designed for recording scalp and skin hemodynamic interference. Far channels were formed from source-detector pairs from neighboring modules. Among the modules, 16 served as sources and the remaining 16 as detectors for far channels, establishing 51 far channels through nearest module source-detector pairings. Data acquisition was conducted at a sampling rate of 1.58 Hz. During measurements, participants were instructed to sit comfortably in a chair with their eyes closed for 5 min before and after each intervention session.

### fNIRS preprocessing and analysis

HOMER software was used for fNIRS processing, which involved converting intensity changes to optical density changes and resolving relative HbO and Hb changes in the short- and far-separation channels [27]. Each channel was detrended by subtracting the mean of the data set from all points. The channel was then bandpass filtered using frequencies ranging from 0.01 to 0.1 Hz. Motion artifacts in individual channels were corrected using the hmrMotionCorrectWavelet function in HOMER (interquartile range [IQR] = 0.75) [28–30]. The influence of superficial hemodynamics was minimized by regressing the data collected from the short-separation channel within each module from the nearest neighboring far channel. The nearest-neighbor approach reportedly removes superficial signals more effectively due to inhomogeneous systemic interference [31]. Individual channels were rejected due to excessively high or low standard deviation (i.e. >95% or < 5% percentile of all channels’ standard deviation). Due to the high intertrial variability of tDCS, the analysis was performed by averaging the hemodynamic changes across 10 trials for each participant to reduce individual-trial noise, such as tDCS stimulation setting, circadian rhythms, psychological and physiological conditions, and the variability of the injured brain [32, 33].

Channel data were aligned among all participants to generate lesion and nonlesion channels for group analysis.

DOT images were analyzed using a sensitivity matrix calculated using AtlasViewer [34]. Anatomically guided image reconstruction was performed using the Colin27 MRI template, and modules were anchored to specific 10 − 20 positions. Linear DOT images were analyzed using spatially variant regularization [35]. For further anatomical localization, each DOT image was divided into three regions (frontal, motor, and parietal) on both lesion and nonlesion sides. By translating the 10–20 position to the Brodmann area (BA), the frontal, motor, and parietal regions were identified to encompass BA-8, BA-4, and 6, and BA-5, 39, and 40, respectively [36].

**Fig. 3:**
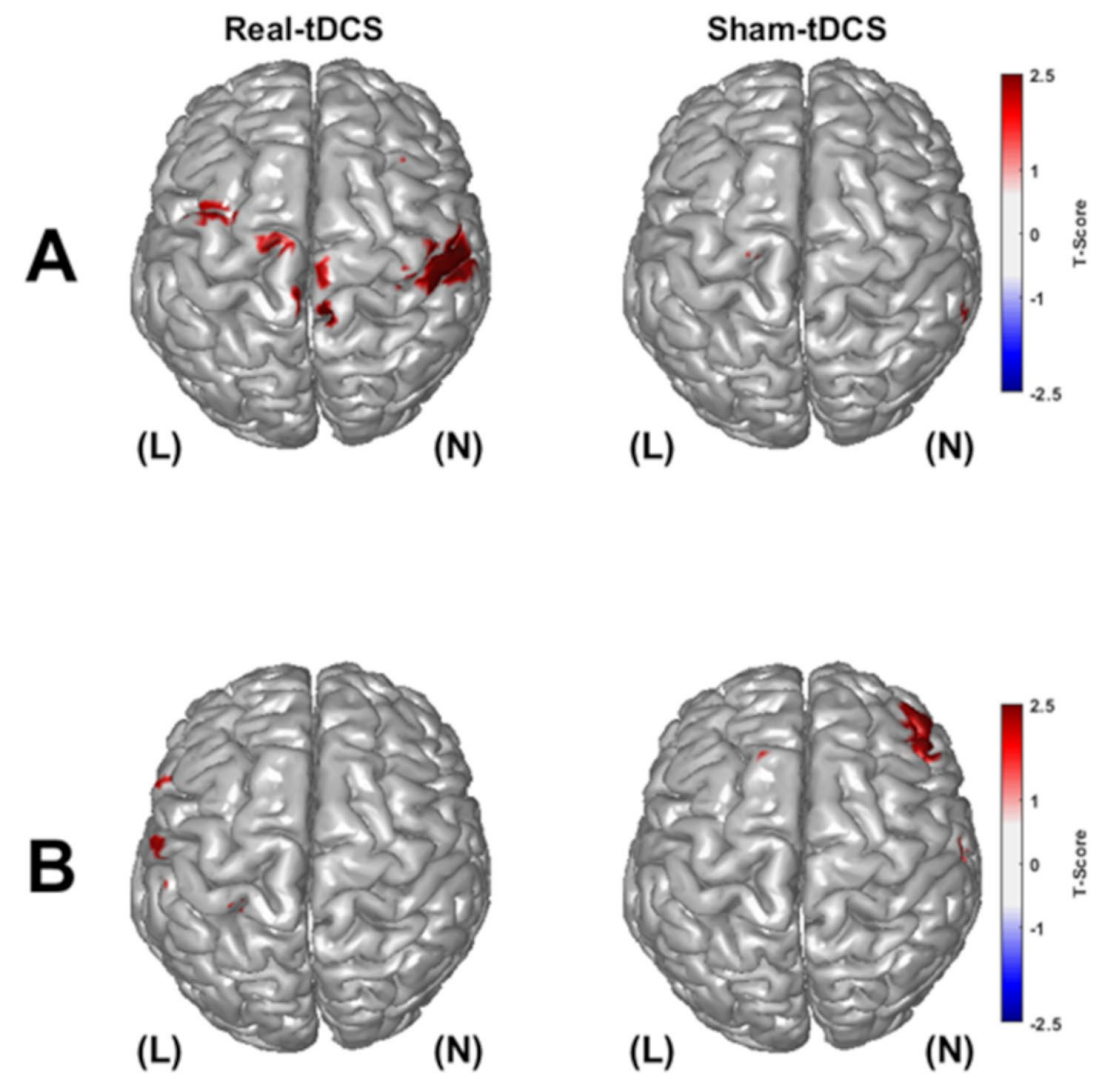
T-maps of significant changes of **(A)** accumulated HbO and **(B)** accumulated Hb changes from prestimulation to poststimulation for real-tDCS (left) and sham-tDCS (right) participant groups

HbO_Acc_ and Hb_Acc_ were employed to measure the resting-state net change in cerebral oxygenation by integrating the time-series data of HbO and Hb. The numerical integration of the time series was conducted using the trapz function in MATLAB (MathWorks, Natick, MA). This function approximates the area under the curve of time-series data by dividing the signal into trapezoids and summing their areas [37]. The integration of time series hemodynamics has been used to quantify sustained hemodynamics during clinical procedures and to distnguish between rest and task states [38–40]. Supplementary Fig. 3 illustrates an example of HbO_Acc_ calculation for the first 60 s of patient data. According to Fig. 3A, a substantial decrease in HbO led to a net decrease in HbO_Acc_ during this period; according to Fig. 3B, an increase in HbO led to a net increase in HbO_Acc_. Hence, HbO_Acc_ and Hb_Acc_ calculations are potentially useful in assessing net changes of resting-state cerebral hemodynamic responses to tDCS.

Additionally, to compare pre and post-HbO_Acc_ and Hb_Acc_, a two-sided t-tail test was conducted for each vertex in the DOT group, encompassing all real and sham participants. The t-values were assigned to vertices to create a t-map, highlighting significant areas of hemodynamic accumulation. Seeded connectivity networks were also calculated as an additional method for assessing resting-state hemodynamic changes. As discussed earlier, six regions of interest (ROIs) were formed based on BA position. Within each ROI, the average of resting-state time-series fluctuations was calculated to derive the seed signal. Subsequently, the Pearson correlation coefficient of the seed signal and every vertex in the DOT brain was calculated to generate seeded connectivity networks.

### Upper limb functional assessments and neurophysiological assessment

Functional motor changes were assessed using the Fugl–Meyer Assessment for Upper Extremity (FMA-UE), which has a score range of 0–66. The FMA-UE is a comprehensive and quantitative tool for assessing motor function, widely acknowledged for its effectiveness in evaluating motor recovery in patients with stroke [41]. To assess gross motor function, the Box and Block Test (BBT) was employed. This test measures the ability to transport blocks from one compartment to another within 60 s [42]. Finger dexterity was assessed using a nine-hole pegboard test, which measures the time taken for a patient to insert pegs into all holes on the board [43]. Grip strength and tip-pinch strength measurements were conducted to determine the maximum force exerted by a patient’s forearm and fingertips, respectively [44]. In addition, fine motor control was further assessed using the Sequential Finger Tapping Test (SFTT), which evaluates the speed and accuracy of sequential finger movements [9]. Overall hand function was assessed through the Jebsen-Taylor Hand Function Test (JTHFT), which involves a series of tasks mimicking everyday hand activities [45]. Furthermore, Motor Evoked Potential (MEP) measurements were performed to evaluate neurophysiological responses, providing insight into corticospinal excitability and motor pathway integrity [46]. These functional tests have been validated as accurate methods for assessing motor function improvement in patients with stroke undergoing tDCS interventions [47, 48].

### Statistical analysis

Statistical analyses were performed using IBM SPSS Statistics for Windows, version 20.0 (IBM Corp., Armonk, NY, USA). The Shapiro–Wilk test and Levene’s test were employed to evaluate the normality of the data and the homogeneity of variances, respectively. Parametric analyses were performed for data meeting normality criteria, while nonparametric analyses were applied otherwise. Differences between groups at pre and poststimulation were assessed using the independent t-test for continuous variables and the chi-square test for categorical variables. Multiple comparison correction was performed using Benjamini and Hochberg false discovery rate control procedure (α = 0.05) [49, 50]. 

To analyze improvements in upper limb function attributed to multi-channel tDCS, a two-way repeated measures ANCOVA (analysis of covariance) was conducted, with stroke type included as a covariate to adjust for baseline differences between groups. The analysis focused on the group × time interaction, evaluated using repeated measures ANCOVA, to determine the effects of the intervention across different time points (before the intervention, T0, and after 10 intervention sessions, T1). Additionally, to investigate the effects of multi-channel tDCS on cerebral hemodynamic, fNIRS data (HbOAcc and HbAcc) were analyzed using two-way repeated measures ANCOVA, with stroke type was included as a covariate to adjust for baseline differences between groups. The analysis focused on the group × time interactions to evaluate the effects of the intervention over time (before the intervention, T0 and after 10 intervention sessions, T1). Pearson’s correlation coefficients were calculated to investigate the relationships between changes in motor function and HbO_Acc_ from the fNIRS analysis.

## Results

### Changes in accumulated hbO and hb after multi-channel tDCS stimulation

To measure areas of significant hemodynamic accumulation, HbO_Acc_ and Hb_Acc_ t-scores were calculated by comparing pre and poststimulation values among all participants in the real- and sham-tDCS groups. Figure 3 displays the HbO_Acc_ and Hb_Acc_ t-maps for the real- and sham-tDCS groups. The t-maps were thresholded for vertices that tested significantly different (p-value < 0.05) after multiple comparisons correction. After thresholding, the significant areas of activations were in the bi-lateral motor areas in the real-tDCS groups for HbO_Acc_. The sham-tDCS group did not not show significant areas of activiation in the motor area for HbO_Acc_ and Hb_Acc_ when comparing pre-stimulation to post-stimulation.

**Fig. 4:**
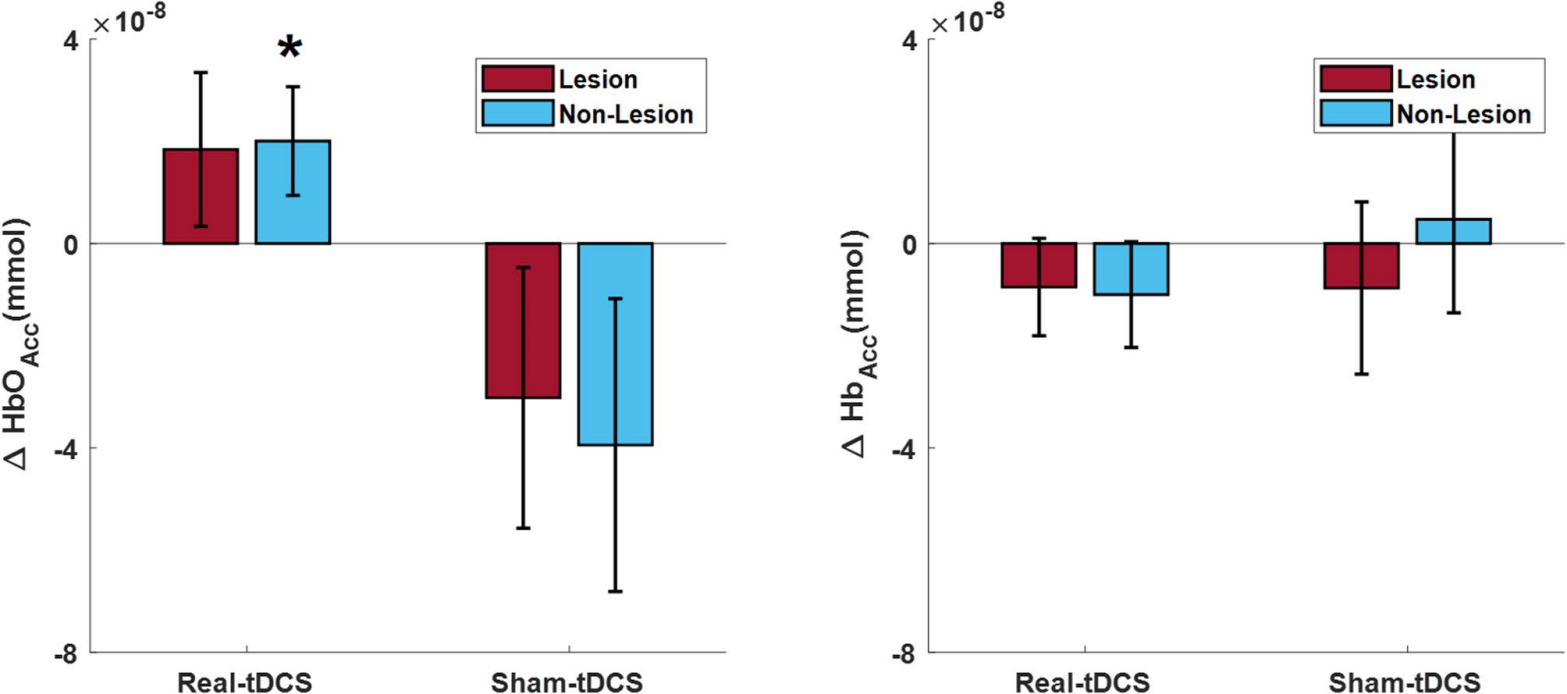
Comparison of accumulated changes in HbO (HbO_Acc_) (left) and Hb (Hb_Acc_) (right) from prestimulation to poststimulation periods for the lesion (red) and nonlesion sides (blue) for real-tDCS and sham-tDCS groups

To quantify HbO_Acc_ and Hb_Acc_ changes, the difference between post and prestimulation accumulated hemodynamics was calculated for each participant and averaged. Figure 4 illustrates the average HbO_Acc_ and Hb_Acc_ changes for the lesion and nonlesion sides in the real- and sham-tDCS groups. The real-tDCS group demonstrated an increase in HbO_Acc_ for both lesion and nonlesion hemispheres, a phenomenon not observed in the sham-tDCS group (Fig. 4). The change in HbO_Acc_ on the nonlesion side was significantly greater in real-tDCS participants compared to sham-tDCS participants (*p* = 0.033). Although a similar trend was observed on the lesion side, the difference between the real- and sham-tDCS groups was not signficant with a *p*-value of 0.058. The real- and sham-tDCS groups did not exhibit significant increases in Hb_Acc_ poststimulation on both the lesion and nonlesion sides.

Supplementary Table 1 shows the changes in hemodynamic response after multi-channel tDCS interventions. The group × time interaction for HbOAcc was statistically significant effects in region 5 (*p* = 0.028). In contrast, no significant the group × time interaction were observed for other regions. For HbAcc, the group × time interaction was significant in region 6 (*p* = 0.031), with the real-tDCS group exhibited a significant reduction in deoxyhemoglobin accumulation after the intervention. In contrast, the sham-tDCS group exhibited no significant changes in HbAcc (*p* = 0.073). The group × time interactions for other regions were not significant. A significant group effect was found for HbOAcc in Region 5 and for HbAcc in Region 6. Additionally, a significant time effect was found for HbAcc in region 6, but no significant effect was observed for HbOAcc.

### Changes in seed-based functional connectivity after multi-channel tDCS stimulation

**Fig. 5:**
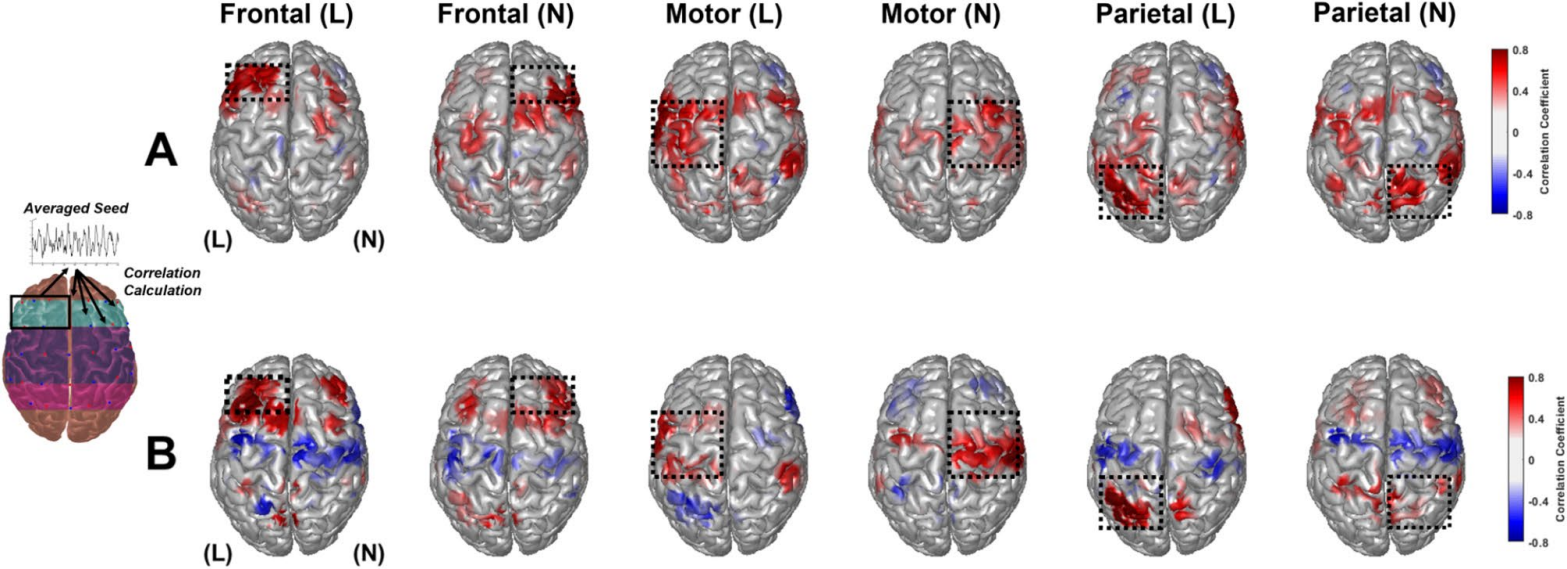
Seeded connectivity analysis of HbO time-series change poststimulation for six regions of interest for **(A)** real-tDCS and **(B)** sham-tDCS groups. The location of the seeds is indicated with a yellow dot on the cortical surface

**Fig. 6:**
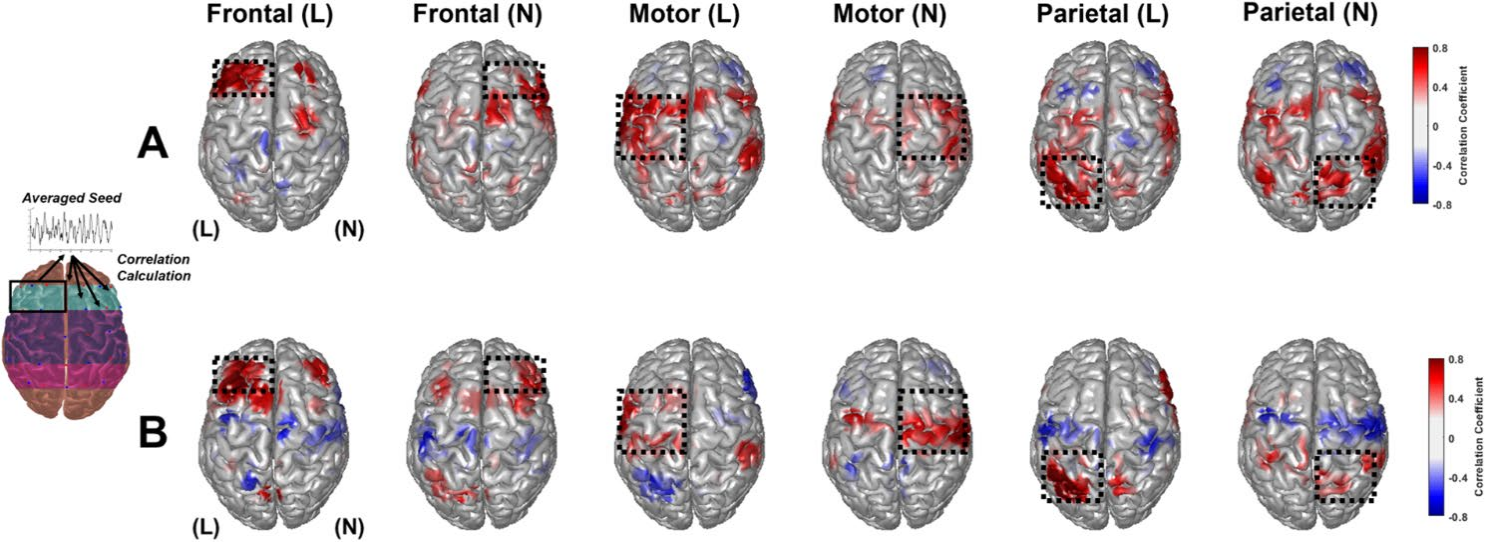
Seeded connectivity analysis of Hb time-series change poststimulation for six regions of interest for **(A)** real and **(B)** sham-tDCS groups. The location of the seeds is indicated with a yellow dot on the cortical surface

Time-varying changes can be assessed using seeded functional connectivity measurements as an alternative method for examining whole-brain network changes caused by direct stimulation. Seeds were calculated for three functional brain areas: frontal, motor, and parietal. Each functional area was evaluated on the lesion and nonlesion sides to form six ROIs. Within each ROI, HbO and Hb time-series poststimulation changes were averaged across the real- and sham-tDCS groups, creating six ROI seeds. Subsequently, for each seed, a correlation coefficient was calculated between the ROI seed and the vertice. Figures 5 and 6 depicts the seeded HbO, Hb connectivity networks based on the six ROI seeds, for the (A) real-tDCS group and (B) sham group.

In the real-tDCS group, lesion and nonlesion frontal seeds exhibited strongly positive correlated hemodynamics concentrated in their respective frontal regions. The sham-tDCS group exhibited the same concentrated connectivity in the frontal region, along with a negative correlation with motor areas on the contralateral side of the seed. For the motor region seeds, the real-tDCS group displayed a broad area of connectivity spanning both hemispheres, particularly evident in the lesion-side motor seed. However, the sham-tDCS group did not demonstrate the same extensive connectivity for the motor seeds. Finally, the parietal seeds displayed a wide area of connectivity in the real-tDCS group across both hemispheres. However, the sham-tDCS group demonstrated considerably lower connectivity and connective dissociation from the brain’s motor area.

Figure 6 illustrates the whole-brain connectivity networks for the six seeds of the Hb time-series data. Similar to the HbO connectivity networks, the real-tDCS group exhibited extensive connectivity, specifically for the motor and parietal seeds. The sham-tDCS group displayed a similar connective disassociation with the motor areas of the frontal and parietal seeds. The strong HbO and Hb connectivity observed in the real-tDCS group, particularly in the motor and parietal regions, indicates that tDCS may induce widespread vascular oscillations in patients with stroke.

### Changes in upper limb function after 10 multi-channel tDCS interventions

The group × time interaction did not show statistically significant differences between the real-tDCS and sham-tDCS groups for the total FMA-UE score or any subscale (two-way repeated measures ANCOVA, with stroke type as a covariate; adjusted *p* = 0.114 for total FMA-UE) (Table 2).

**Table 2:** Changes in Fugl–Meyer assessment for upper limb after multi-channel tDCS interventions

	Real-tDCS		Sham-tDCS		Adjusted *p*-value
	T0	T1	T0	T1	
Total	43.8±12.9	47.4±13.5	37.7±18.5	40.1±18.5	0.114
Subscale					
Shoulder/Elbow/Forearm	28.4±6.4	29.8±6.7	24.4±9.0	25.5±9.7	0.557
Wrist	4.8±0.0	5.4±3.2^*^	3.8±3.5	4.0±3.4	0.093
Hand	7.5±3.8	9.0±4.4	6.7±4.9	7.6±4.8	0.552
Coordination	3.0±1.4	3.3±1.5^*^	2.8±2.3	3.0±2.1	0.267

Values are presented as the mean ± standard deviation. The adjusted *p*-value represents the group × time interaction from the two-way repeated measures ANCOVA, with stroke type as a covariate. T0, before the interventions, and T1, after completing all 10 interventions

The group × time interaction was statistically significant for the BBT (adjusted *p* = 0.004) but not for the other upper limb functional assessment measures (two-way repeated measures ANCOVA, with stroke type included as a covariate) (Table 3). Within-group analyses using ANCOVA revealed that the real-tDCS group showed significant improvements in the BBT (*p* < 0.01), indicating enhanced manual dexterity between T0 and T1.

**Table 3:** Changes in other upper limb functional assessment tests after multi-channel tDCS interventions

	Real-tDCS		Sham-tDCS		Adjusted *p*-value
	T0	T1	T0	T1	
Nine-hole pegboard test (s)	129.7±110.9	114.2±101.4	153.7±130.7	140.3±124.5	0.974
Box and block test (EA)	24.4±16.8	29.9±20.2^**^	26.6±22.1	26.8±22.0	0.004^†^
Grip-strength test (kg)	5.2±7.6	7.0±7.9	5.8±8.0	6.7±9.4	0.155
Tip-pinch strength test (kg)	0.5±0.3	0.4±0.3	0.5±0.5	0.5±0.5	0.553
Sequential finger tapping test					
Accuracy (%)	55.7±29.7	57.8±31.2	35.4±35.7	37.7±40.1	0.423
Response time (msec)	690.1±199.8	654.4±243.7	754.1±228.6	761.4±229.8	0.203
Skill index	9.7±6.5	11.6±8.8	6.3±6.6	6.9±7.9	0.097

Values are presented as the mean ± standard deviation. ^†^, A significant change was identified in the group × time interaction from the two-way repeated measures ANCOVA, with stroke type included as a covariate, *p* < 0.05. ^*^, Significant change between T0 and T1 based on within-group ANCOVA with baseline scores and stroke type as covariates, *p* < 0.05, ^**^, *p* < 0.01. T0, before the interventions, and T1, after completing all 10 interventions

The group × time interaction was not significant for other functional and neurophysiological assessments, including the nine-hole pegboard test, grip strength, tip-pinch strength, and SFTT accuracy, response time, and skill index. Additionally, the Jebsen-Taylor Hand Function Test showed no significant changes across all subcategories (writing, card turning, lifting small objects, feeding, stacking, lifting large light objects, and lifting large heavy objects) (Supplementary Table 2). Similarly, neurophysiological measures, including resting motor threshold, amplitude, and latency, did not exhibit significant changes between T0 and T1 (Supplementary Table 3). No adverse reactions were reported among the 24 participants who completed the study.

### Relationship between changes in upper limb function and accumulated HbO

**Fig. 7:**
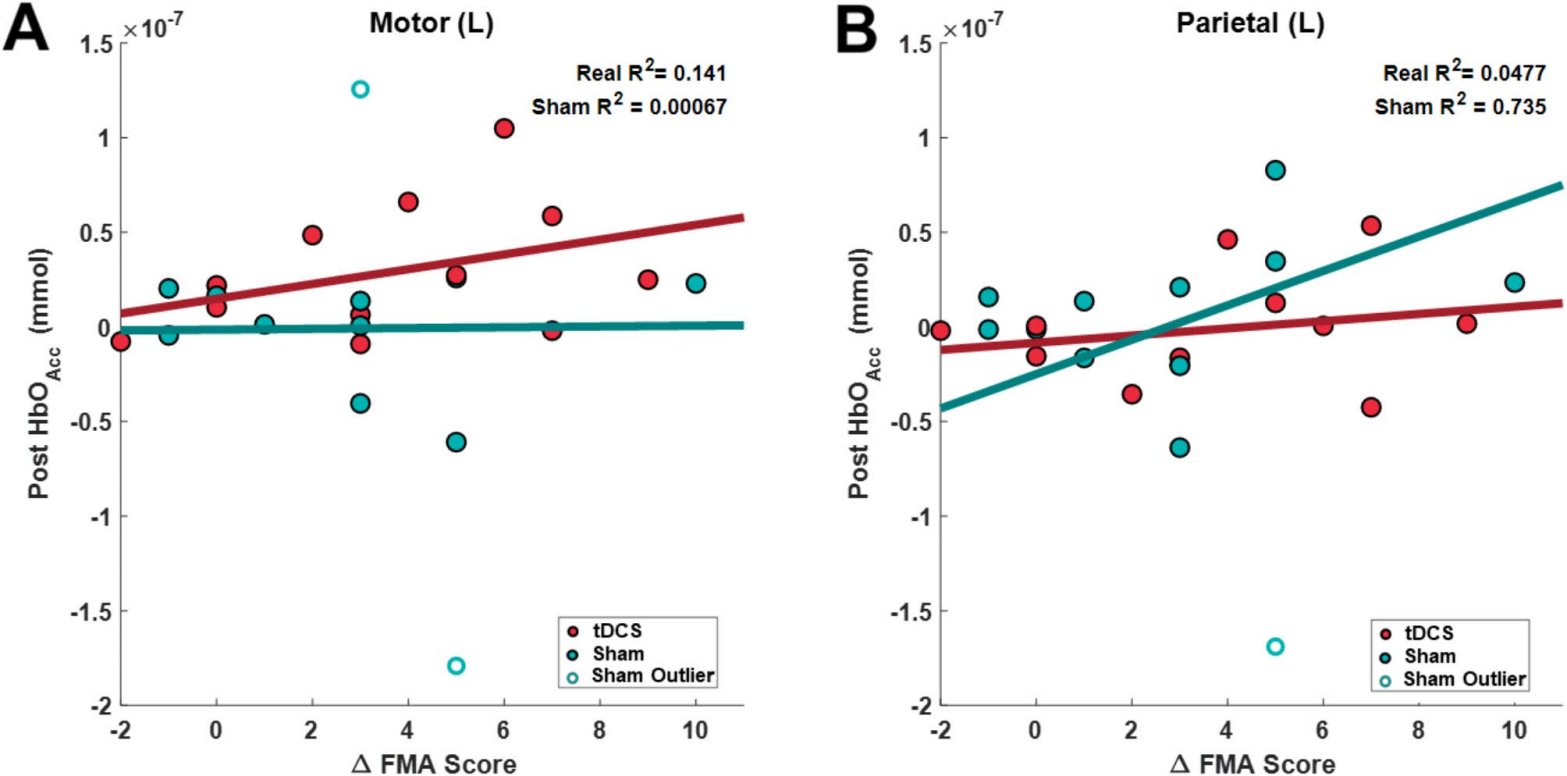
Comparison of accumulated HbO (HbO_Acc_) poststimulation for the lesion-side motor and parietal regions and the change in Fugl-Myer assessment for upper limb score (FMA-UE). Individual participants who received real-tDCS stimulation are depicted with red dots, while those who received sham-tDCS are represented by green dots. **(A)** Outliers in HbOAcc (z-score > 2) are indicated with an outlined circle. The red arrow denotes highly linear changes in HbOAcc and changes in FMA-UE score for six participants who received real-tDCS stimulation. **(B)** Comparison of HbO_Acc_ and change in FMA-UE scores, excluding outliers, with the linear regression line (real-tDCS = red, sham-tDCS = green). The *p*-value of linear regression is reported on the top corner of each region

As revealed by the seeded connectivity analysis (Figs. 5 and 6), which showed the motor and parietal areas with the strongest connectivity measurements, we investigated the correlation between poststimulation HbO_Acc_ and changes in FMA-UE scores for each participant. Figure 7 shows the comparison between changes in FMA-UE score and HbO_Acc_ in the lesion-side motor and parietal regions of individual participants in the real- and sham-tDCS groups. Among all segmented brain regions, the lesion-side motor and parietal sides exhibited the most linear relationship between changes in FMA-UE score and HbO_Acc_. Outliers in the HbO_Acc_ data were identified by performing z-score normalization for all participants (real and sham-tDCS), and points with a z-score > ± 2 were excluded. This method identified two participants from the sham-tDCS group as outliers and removed from linear fitting.

After removing the outliers, linear fitting was performed between the clinical and fNIRS-derived metrics. The goodness of fit (R^2^) for the linear fitting for the tDCS group, sham group and select tDCS subjects are shown in Table 4. The highest R^2^ was seen in sham group in the parietal non-lesion region, indicating a linear relationship between fNIRS metrics and FMA score for these groups. The low R^2^ for the tDCS group in both the motor and parietal regions indicate a nonlinear realtionship between Post HbO_Acc_ and change in FMA scores.

**Table 4:** Goodness-of-Fit for post HbO_Acc_ and ΔFMA score

	Motor (Lesion) *R* ^2^	Parietal (Lesion) *R* ^2^
tDCS (*n* = 12)	0.141	0.0477
Sham (*n* = 10)	0.000109	0.735
Select tDCS (*n* = 6)	0.988	0.122

## Discussion

This study investigated the effects of multi-channel tDCS on the ipsilesional M1 and aIPS in patients with stroke, focusing on altering resting-state hemodynamic responses and improving upper limb motor functions. The real-tDCS group showed a significant increase of HbOAcc from the pre-stimulation period to post-stimulation period in the motor area, along with larger areas of positive connectivity to the motor area. The sham group did not show a signficant change of HbO_Acc_ and Hb_Acc_ from pre-stimulation and post-stimulation, in addition to negative connectivity in the motor area. Inter-domain connectivity due to tDCS stimulation has shown to be a characteristic of enhanced functionality and diminished regional segregation [51]. Conversely, the negative correlation observed in our sham group has also been observed in previous studies, associated with severe motor impairments due to strong regional segregation of brain function [52]. The BBT demonstrated a significant group × time interaction, indicating that the real-tDCS group experienced greater improvements compared to the sham group. While poststimulation changes in HbOAcc were examined in relation to FMA-UE scores, no strong linear relationship was observed in the real-tDCS group. These findings suggest that multi-channel tDCS targeting the ipsilesional M1 and aIPS may have a role in supporting motor recovery. They also emphasize the value of assessing cerebral oxygenation changes in understanding the recovery processes.

Noninvasive brain stimulation, such as tDCS, is known to induce vasodilation in response to increased neural metabolism. However, the broader effects on cerebral oxygenation are less explored [19, 32, 53]. The post-tDCS in a resting state is known to induce cortical activation [54], facilitating sufficient blood perfusion in the brain to meet the increased oxygen demand [55]. This process provides essential evidence for exploring the impact of tDCS on cerebral hemodynamic responses and its therapeutic potential. In this context, our study concentrated on the accumulation of hemodynamics during resting-state periods before and after stimulation to examine the impact of tDCS on cerebral blood flow. The findings unveil a significant increase in HbO_Acc_ within the motor cortex on the lesioned side in the real-tDCS group poststimulation, a pattern not mirrored in the sham-tDCS group. This was accompanied by a decrease in Hb_Acc_, indicating enhanced blood flow and hemoglobin clearance, indicative of a vasodilatory response to the increased metabolic demand [56, 57].

Impaired cerebral perfusion, particularly in older or stroke-affected patients, can contribute to cognitive and motor deficits [58, 59]. Our results showed dissociation in connectivity between motor, frontal, and parietal regions in the sham group, whereas the real-tDCS group exhibited enhanced connectivity, especially in the motor cortex of the lesioned side, and improvements in FMA_UE scores, aligning fNIRS metrics with clinical motor recovery evaluations. While sham participants displayed minor FMA score improvements, the increase in parietal HbO_Acc_ suggests partial motor recovery, emphasizing the potential of fNIRS to complement conventional clinical assessments and mitigate potential biases [60].

The group × time interaction was statistically significant for the BBT, indicating that the real-tDCS group demonstrated greater improvements in gross manual dexterity compared to the sham group. This finding supports the potential superiority of real tDCS over sham in enhancing specific aspects of motor function. These findings align with an uncontrolled pilot study where 4 × 1 multi-channel tDCS targeting the ipsilesional M1 at 1 mA for 20 min across four days resulted in notable improvements in gross motor function, including FMA-UE and BBT scores [17]. Similarly, a randomized controlled trial focusing on ischemic stroke patients found that 2 mA stimulation of the ipsilesional M1 for 20 min reduced the latency of M1 motor-evoked potentials and increased FMA-UE scores relative to sham stimulation [10]. Collectively, these results indicate that multi-channel tDCS shows potential in supporting motor recovery, though further research is required to clarify its efficacy across different motor domains. While the significant group × time interaction observed for the BBT indicates potential benefits, the findings for other outcomes, such as FMA-UE, require cautious interpretation due to the lack of significant interaction effects. Targeting the M1 and aIPS regions, when combined with rehabilitation exercises, may represent a complementary approach for stroke rehabilitation, but further research is needed to confirm these effects. The supplementary results provide additional insights, showing no significant changes in fine motor tasks, such as the Jebsen-Taylor Hand Function Test, or in neurophysiological outcomes, such as resting motor threshold and motor-evoked potential amplitude. These findings suggest that while multi-channel tDCS may impact gross motor function, its effects on fine motor control and neurophysiological responses require further investigation.

The results of the two-way repeated measures ANCOVA demonstrate that multi-channel tDCS targeting the ipsilesional primary motor cortex and anterior intraparietal sulcus significantly enhanced cerebral oxygenation and upper limb motor function in stroke patients. The significant Group × Time interaction for both HbO and HbR suggests that tDCS promoted improved cerebral oxygenation, particularly in the motor cortex, which correlated with functional motor improvements. These findings underscore the potential of tDCS as a neuromodulatory intervention to facilitate stroke rehabilitation through both neural and hemodynamic mechanisms.

The simultaneous stimulation of both the ipsilesional M1 and the aIPS regions shows potential for supporting motor recovery. The M1 area is traditionally associated with motor control and recovery [61], while the aIPS is associated with spatial awareness and the integration of sensory inputs, crucial for motor planning and execution [62]. Targeting these areas concurrently acknowledges the complex, interconnected nature of motor function recovery. This process relies not only on muscle reactivation but also on cognitive processes, such as attention, planning, and spatial coordination [63]. By stimulating the aIPS alongside the M1, this approach aims to facilitate a more holistic recovery process, potentially leading to more substantial improvements in motor function. While this study does not directly compare stimulation of M1 alone versus M1 plus aIPS, the results hint at the potential benefits. They suggest that targeting not just the primary ROI but also stimulating additional areas related to motor recovery when using multi-channel tDCS could be advantageous. This suggests the viability of employing diverse stimulation strategies with multi-channel tDCS to enhance the efficacy of interventions, offering a broader spectrum of approaches for improving motor recovery in stroke rehabilitation.

This study has several limitations. First, the small sample size and variability in stroke pathology among participants may limit the generalizability of our findings. While improvements were observed in certain measures, such as BBT, these findings should be interpreted with caution. Future studies with larger sample sizes and more robust designs are needed to confirm the efficacy of multi-channel tDCS and its clinical applicability.

Second, the study focused exclusively on ipsilesional stimulation and did not explore contralesional or bilateral stimulation, which may offer additional therapeutic benefits [32, 53]. Including a more diverse participant group and testing various stimulation protocols in future studies would provide a more comprehensive understanding of tDCS’s efficacy.

Third, variability in weekly session frequency due to individual scheduling constraints may have influenced the outcomes. Standardizing session frequency or systematically assessing its impact in future studies could yield clearer insights into the role of intervention frequency.

Lastly, fNIRS measurements were conducted before and after tDCS due to equipment constraints, preventing simultaneous monitoring of hemodynamic responses during stimulation. Future studies should aim to integrate real-time fNIRS and tDCS measurements to better understand the cerebral effects of tDCS. Additionally, excluding patients with M1 or aIPS lesions minimized variability but introduced a selective bias. As many stroke patients have M1 infarctions associated with motor impairments, future studies should include such populations to evaluate the feasibility and effectiveness of tDCS in a broader clinical context.

## Conclusions

This study examined the effects of multi-channel tDCS targeting the ipsilesional M1 and aIPS on upper limb motor function and resting-state cerebral hemodynamics in stroke patients. The intervention was associated with significant improvements in manual dexterity, while fNIRS measurements provided insights into cerebral hemodynamic changes following tDCS. These findings highlight the potential of multi-channel tDCS as a complementary approach in stroke rehabilitation. However, further research with larger sample sizes and optimized stimulation protocols is needed to establish its efficacy more definitively.

## Electronic supplementary material

Below is the link to the electronic supplementary material.

Supplementary Material 1

## Data Availability

The data supporting the conclusion of this article will be made available by the corresponding author upon reasonable request. Prior consent from the participants and necessary approval from the authors’ institutional committees will be required to obtain the data.
