# Supplementary material for: Resting-state hemodynamic changes and effects on upper limb function after multi-channel transcranial direct current stimulation to the ipsilesional primary motor cortex and anterior intraparietal sulcus in stroke patients: an fNIRS pilot study
Source: J Neuroeng Rehabil. 2025 Apr 16;22:83. doi: 10.1186/s12984-025-01618-8 (PMC12001566; doi:10.1186/s12984-025-01618-8)
Supplement: Supplementary file 1 — Supplementary Material 1 [file 12984_2025_1618_MOESM1_ESM.docx]

**Supplementary Figure 1. The CONSORT flow chart**


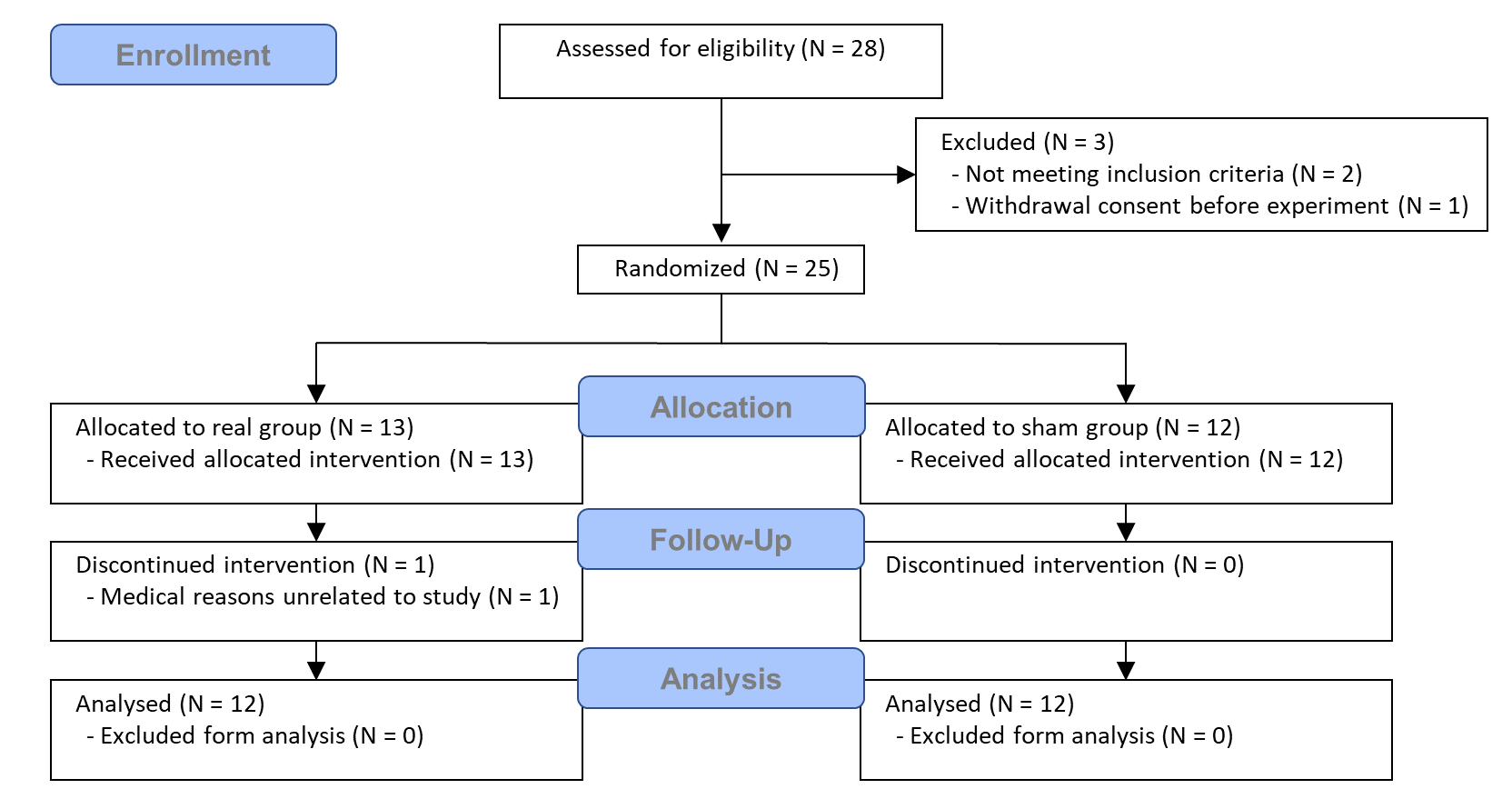


**Supplementary Figure 2. System diagram of proposed integrated tDCS-fNIRS modular system implementation**


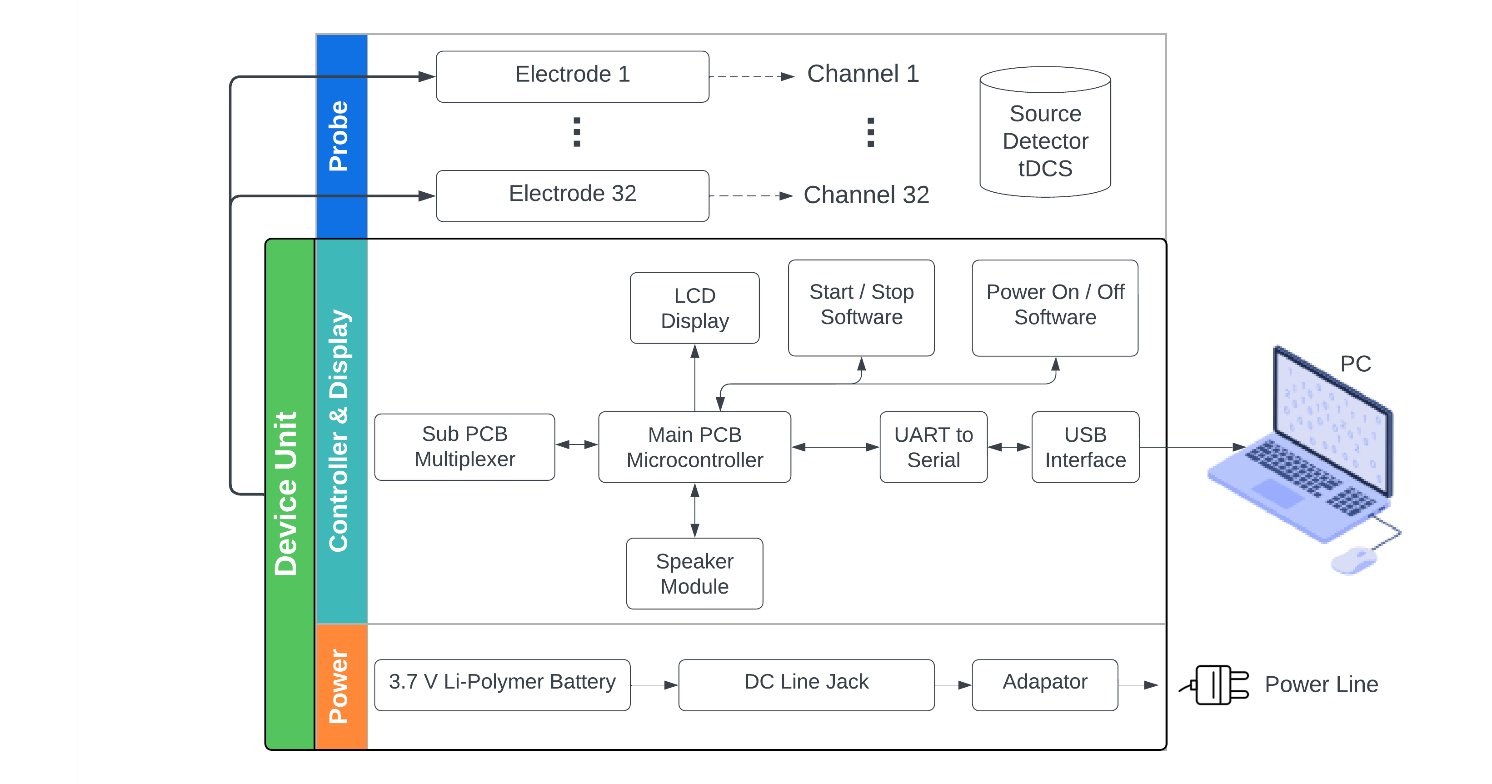


**Supplementary Figure 3. Example of numerical integration to assess accumulated oxy-hemoglobin (HbO_Acc_) from 1 min of resting-state time-series data (shaded areas). (A) a net decrease of HbO_Acc_ (B) a net increase of HbO_Acc_**

**Supplementary Table 1. Changes in hemodynamic response after multi-channel tDCS intervention**

|  | **Regions** | **Group** | **Time** | **group** $\boldsymbol{\times}$ **time**  **(T0 and T1)** |
| --- | --- | --- | --- | --- |
| HbO_Acc_ | Left MFG | 0.894 | 0.675 | 0.821 |
|  | SFG | 0.091 | 0.254 | 0.392 |
|  | Right MFG | 0.712 | 0.924 | 0.886 |
|  | Left SMG | 0.605 | 0.232 | 0.995 |
|  | SPL | 0.033^*^ | 0.11 | 0.028^†^ |
|  | Right SMG | 0.077 | 0.051 | 0.162 |
| Hb_Acc_ | Left MFG | 0.825 | 0.74 | 0.521 |
|  | SFG | 0.083 | 0.362 | 0.448 |
|  | Right MFG | 0.538 | 0.572 | 0.995 |
|  | Left SMG | 0.249 | 0.224 | 0.957 |
|  | SPL | 0.216 | 0.073 | 0.053 |
|  | Right SMG | 0.006^**^ | 0.013^*^ | 0.031^†^ |

Values are presented as the *p*-value. ^†^, A significant change was identified in the group $\times$ time interaction from the two-way repeated measures ANCOVA, with stroke type included as a covariate, p < 0.05. ^*^, Significant difference between real-tDCS and sham-tDCS groups, based on between-group ANCOVA with stroke type as covariates, *p* < 0.05, ^**^, *p* < 0.01. T0, before the interventions, and T1, after completing all 10 interventions, MFG; middle frontal gyrus, SFG; superior frontal gyrus, SMG; supramarginal gyrus, SPL; superior parietal lobule.

**Supplementary Table 2. Changes in Jebsen-Taylor hand function test after multi-channel tDCS interventions**

|  | Real-tDCS | | Sham-tDCS | | Adjusted  *p*-value |
| --- | --- | --- | --- | --- | --- |
|  | T0 | T1 | T0 | T1 |  |
| Writing | 71.5$\pm$51.3 | 65.1$\pm$37.2 | 63.9$\pm$42.2 | 66.7$\pm$41.6 | 0.119 |
| Card turning | 30.7$\pm$41.9 | 32.4$\pm$41.3 | 50.7$\pm$53.0 | 53.2$\pm$51.1 | 0.456 |
| Lifting small objects | 53.2$\pm$44.3 | 43.8$\pm$40.1 | 62.9$\pm$52.3 | 64.3$\pm$50.9 | 0.161 |
| Feeding | 41.2$\pm$42.1 | 42.5$\pm$44.8 | 52.4$\pm$50.7 | 48.4$\pm$52.9 | 0.391 |
| Stacking | 30.2$\pm$42.8 | 29.7$\pm$42.8 | 45.6$\pm$55.0 | 44.4$\pm$55.8 | 0.938 |
| Lifting large light objects | 30.8$\pm$41.9 | 33.1$\pm$42.7 | 59.3$\pm$60.4 | 50.0$\pm$53.1 | 0.418 |
| Lifting large heavy objects | 30.4$\pm$42.3 | 30.7$\pm$42.8 | 48.1$\pm$53.6 | 47.4$\pm$54.0 | 0.585 |

Values are presented as the mean ± standard deviation. Unit, sec. The adjusted *p*-value represents the group $\times$ time interaction from the two-way repeated measures ANCOVA, with stroke type as a covariate. T0, before the interventions, and T1, after completing all 10 interventions.

**Supplementary Table 3. Changes in neurophysiological response after multi-channel tDCS interventions**

|  | Real-tDCS | | Sham-tDCS | | Adjusted  *p*-value |
| --- | --- | --- | --- | --- | --- |
|  | T0 | T1 | T0 | T1 |  |
| Resting motor threshold (rMT, %) | 55.4$\pm$9.9 | 53.6$\pm$9.1 | 57.4$\pm$14.0 | 57.6$\pm$13.0 | 0.346 |
| Amplitude ($\mu$V, 120% rMT) | 410.6$\pm$306.3 | 298.6$\pm$285.8 | 211.1$\pm$224.6 | 269.7$\pm$233.7 | 0.087 |
| Latency (msec, 120% rMT) | 24.0$\pm$5.1 | 25.5$\pm$5.3 | 27.6$\pm$8.9 | 22.0$\pm$6.3 | 0.209 |

Values are presented as the mean ± standard deviation. The adjusted *p*-value represents the group $\times$ time interaction from the two-way repeated measures ANCOVA, with stroke type as a covariate. T0, before the interventions, and T1, after completing all 10 interventions.
